# Urinary quality of life in patients treated with prostate SBRT with intra-prostatic boost

**DOI:** 10.3389/fonc.2025.1622191

**Published:** 2025-09-30

**Authors:** Nisha Bhargava, Martina Hurwitz, Josephine Levey, Lily Bennett, Joseph A. Aronovitz, Daniel R. Schmidt, Liana Dawson, Jonathan W. Lischalk, Irving D. Kaplan, Nima Aghdam

**Affiliations:** ^1^ Department of Radiation Oncology, Beth Israel Deaconess Medical Center, Boston, MA, United States; ^2^ Department of Radiation Oncology, Brigham and Women’s Hospital, Boston, MA, United States; ^3^ Radiation Oncology, New York University (NYU) Grossman Long Island School of Medicine, Mineola, NY, United States; ^4^ Department of Radiation Medicine, MedStar-Georgetown University Hospital, Washington, DC, United States; ^5^ Radiation Oncology, NYCyberknife at Perlmutter Cancer Center -Manhattan, New York, NY, United States

**Keywords:** SBRT (stereotactic body radiation therapy), prostate, QoL (QoL), dominant intraprostatic lesion, dose escalation

## Abstract

**Purpose/objectives:**

SBRT is a standard of care treatment for localized prostate cancer. Whole gland dose escalation remains controversial. Concomitant intraprostatic boost (IPB) may offer an acceptable compromise for dose escalation. In this series, we report changes in International Prostate Symptom Scores (IPSS) over a 12-month period following SBRT with IPB in patients treated in a large academic institution.

**Materials/methods:**

Seventy-four patients treated from October 2018 to March 2022 with robotic stereotactic body radiotherapy completed IPSS questionnaires. IPSS were evaluated for patients at three timepoints: pre-treatment, post-treatment (defined as 3 months after SBRT completion), and at follow-up (defined as within 12 months after SBRT completion). The patients were stratified into two cohorts: patients who experienced minimally important difference (MID) in their post-treatment IPSS and those who did not. Urethral and bladder doses were retrospectively extracted from the treatment planning software and compared between the two cohorts using Wilcoxon rank sum test.

**Results:**

Of the 74 patients, 46 (62%) experienced MID in scores (cohort A), while 28 (38%) did not (cohort B). Patient characteristics in the two cohorts such as risk stratification and initial PSA were well-balanced. Median IPSS for cohort A were 5 (range: 0–21) pre-treatment, 12 (range: 3–28) post-treatment, and 8 (range: 1–32) at 12 months. For cohort B, the scores were 9.5 (range: 0–29), 7 (range: 1–19), and 8.5 (range: 0-32), respectively. In addition, there was a statistically significant difference in D0.03cc to the bladder in cohort A compared to cohort B (41.9 Gy vs 40.2 Gy; p < 0.001).

**Conclusion:**

IPB is well tolerated with acceptable change in urinary quality of life metrics as measured by IPSS. Max dose to the bladder remains the only significant difference in patients who experienced MID in their urinary quality of life.

## Introduction

Stereotactic body radiotherapy (SBRT) has become a standard treatment for localized prostate cancer, offering favorable disease control with a hypofractionated schedule (1). This approach significantly reduces treatment duration while maintaining outcomes comparable to conventional fractionation regimens. However, SBRT necessitates precise dosimetric planning to limit dose exposure to adjacent organs at risk (OARs), particularly the bladder and urethra. Excess dose to these structures has been linked to acute urinary symptoms, adversely affecting patient-reported quality of life (QoL) (2).

Intraprostatic boost (IPB) has emerged as a promising method to selectively escalate dose delivery to dominant intraprostatic lesions (DILs) identified on multiparametric MRI. Pucar et al. demonstrated that locally recurrent prostate tumors align with the DIL, suggestive of a need for dose escalation to these lesions which harbor significant disease (3). Additionally, on retrospective analysis, Gorovets et al. found that PI-RADS 4 or 5 DIL on pre-treatment MRI was associated with positive biopsies after treatment (4). The FLAME trial demonstrated that focal dose escalation to DILs significantly improves biochemical disease-free survival without increasing toxicity, providing robust evidence for partial gland dose escalation (5). Furthermore, DELINEATE trial also shows acceptable toxicity in the hypo fractionated setting (6). Employing IPB would also enhance the biological efficacy of SBRT while sparing surrounding OARs. Hypo-FLAME results supports the feasibility of IPB with excellent biochemical control without significantly increasing treatment-related toxicity (7). Moreover, in a small phase II study, Yasar et al. demonstrated the feasibility of SBRT delivered with real-time fiducial tracking with dose escalation to the DIL, with acute GU toxicity rates that are comparable to ultrahypofractionated SBRT without focal boost (8).

This study investigates the interplay between urethral and bladder dose metrics and their impact on urinary QoL, as measured by longitudinal changes in International Prostate Symptom Scores (IPSS) over 12 months in patients treated with SBRT and IPB.

## Method

This retrospective study included 74 patients treated with robotic SBRT with IPB for localized prostate cancer at a single academic institution between October 2018 and March 2022. All patients had a diagnostic staging MRI prior to treatment. Patients were simulated with a contrast enhanced suprapubic urethrogram with 7 cc of iodinated die diluted with a 17 mL mixture of viscous lidocaine and 5-10 cc of normal saline to aid in delineating the urethra. Dominant intraprostatic lesions (DIL) were delineated using diagnostic and treatment planning MRI relying primarily on T2W and DWI sequences. The planning target volume (PTV) was defined as the prostate volume (GTV) plus proximal seminal vesicles (CTV) with a 5 mm expansion in all directions except posteriorly toward the rectum, where the expansion was 3 mm. Patients received the prescription dose of 36.25 Gy to the PTV and 40 Gy to the GTV. DIL received a median dose of 44 Gy. A separate recently published report describes the dosimetric considerations of this approach. 18 Institutional dose constraints were utilized for evaluation of treatment plans. IPB coverage was limited to meet the OAR constraints. All patients were treated on a CyberKnife M6 platform utilizing multileaf collimation, with inter- and intra-fractional corrections for prostate motion based on tracking 3-4 fiducial markers in the prostate.

Patient-reported IPSS were collected at three time points: pre-treatment, 3 months post-treatment, and 12 months post-treatment. Patients were stratified into two cohorts based on whether they experienced a minimally important difference (MID) in IPSS post-treatment. This was defined as ½ standard deviation of the baseline scores for all patients in this cohort. This is particular definition has been utilized on several studies related to post-SBRT urinary QOL (9–11) and is felt to be robust in diverse populations and settings (12). Dosimetric parameters, including bladder D0.03cc, D0.3cc, V37Gy, and urethral D0.03cc, D0.3cc, V40Gy, were extracted from the treatment planning system. Statistical analysis was conducted using Wilcoxon Rank Sum test, with significance set at p < 0.05. MATLAB R2022b was used for all analyses.

## Results

The 74 patients analyzed had a mean age of 68.9. The initial IPSS median was 6 with a range of 0-29. This median increased to 11 post-treatment with a range of 1-32, but decreased at follow-up to 8 with a range of 0-32. Patient characteristics of the full patient population can be seen in **Table 1**. IPSS were further analyzed based on MID. MID was defined as an increase in score of half the standard deviation of the initial score distribution.

**Table 1:** Patient clinical characteristics for cohorts with and without MID.

Baseline	All Patients	Cohort A (MID)	Cohort B (No MID)
Number of Patients (n)	74	46	28
Mean age in years (SD)	68.9 (5.6)	68.3 (4.9)	69.8 (6.7)
Risk Stratification	n (%)	n (%)	n (%)
Low-risk	2 (2.7)	1 (2.2)	1 (3.6)
Favorable Intermediate-risk	35 (47)	20 (43)	15 (54)
Unfavorable Intermediate-risk	28 (38)	20 (43)	8 (29)
High-risk	9 (12)	5 (11)	4 (14)
Hormonal Therapy	n (%)	n (%)	n (%)
Yes	44 (59)	31 (67)	13 (46)
No	30 (41)	15 (33)	15 (54)
iPSA (ng/mL)			
Mean (SD)	7.88 (3.64)	7.81 (3.52)	8.00 (3.89)
Prostate Volume (cc)			
Median (range)	30.2 (13.0-88.2)	29.4 (13.0-88.2)	30.8 (15.5-76.3)
IPSS Median (range)			
Initial	6 (0-29)	5 (0-21)	9.5 (0-29)
Post-Treatment	11 (1-32)	12 (3-28)	7 (1-19)
Follow-up	8 (0-32)	8 (1-32)	8.5 (0-32)

**Figure 1:**
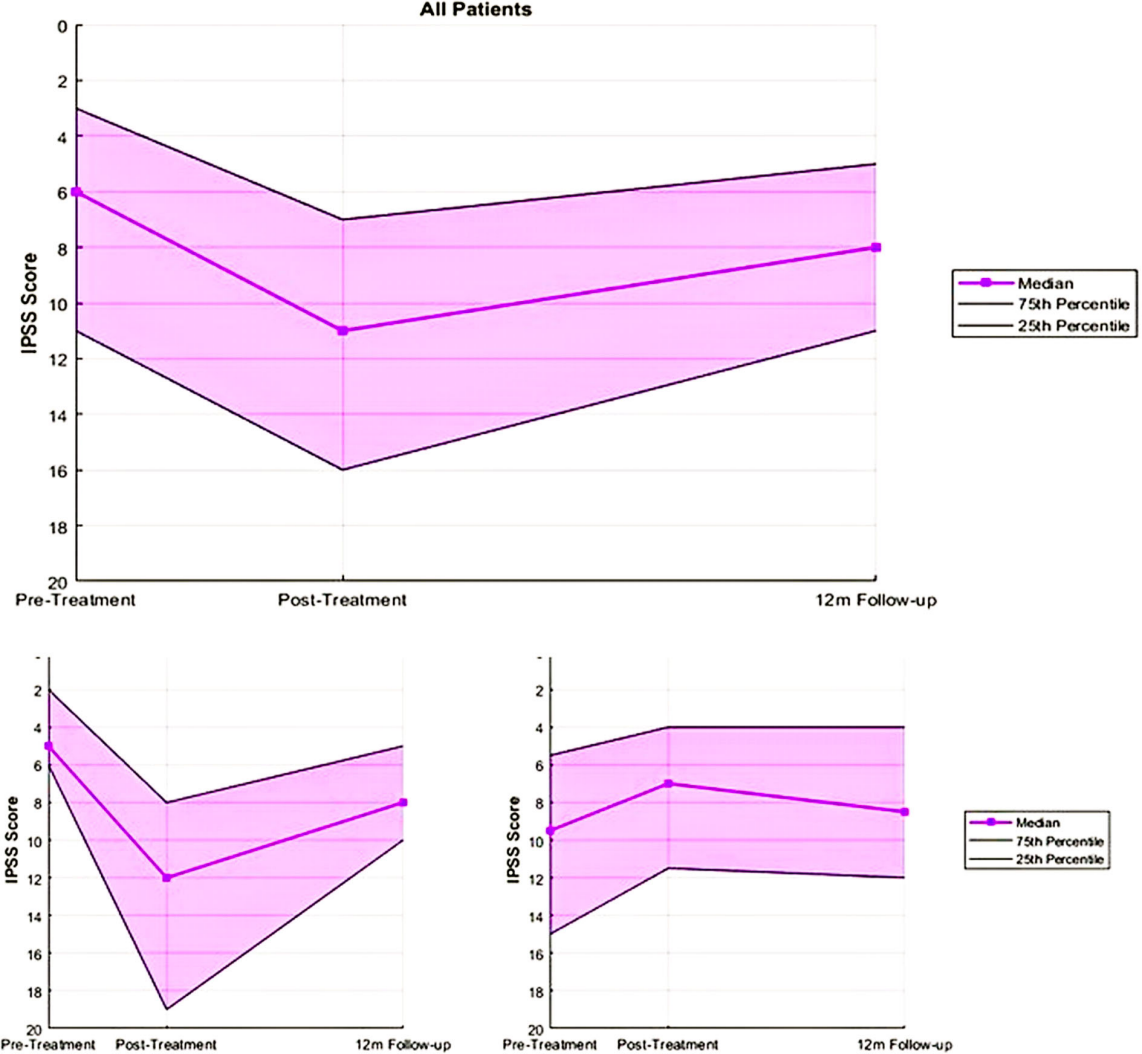
IPSS summary for all patients and cohorts A (those who experienced MID) and B (Those who did not experience MID).

Among the 74 patients, 46 (62%) experienced MID in IPSS (cohort A), while 28 (38%) did not (cohort B). Patient characteristics in the two cohorts such as risk stratification and initial PSA were well-balanced, as seen in **Table 1**. Median scores for cohort A were 5 (range: 0–21) pre-treatment, 12 (range: 3–28) post-treatment, and 8 (range: 1–32) at 12 months. For cohort B, the scores were 9.5 (range: 0–29), 7 (range: 1–19), and 8.5 (range: 0-32), respectively. These scores can be seen in **Figure 1**, below.

**Figure 2:**
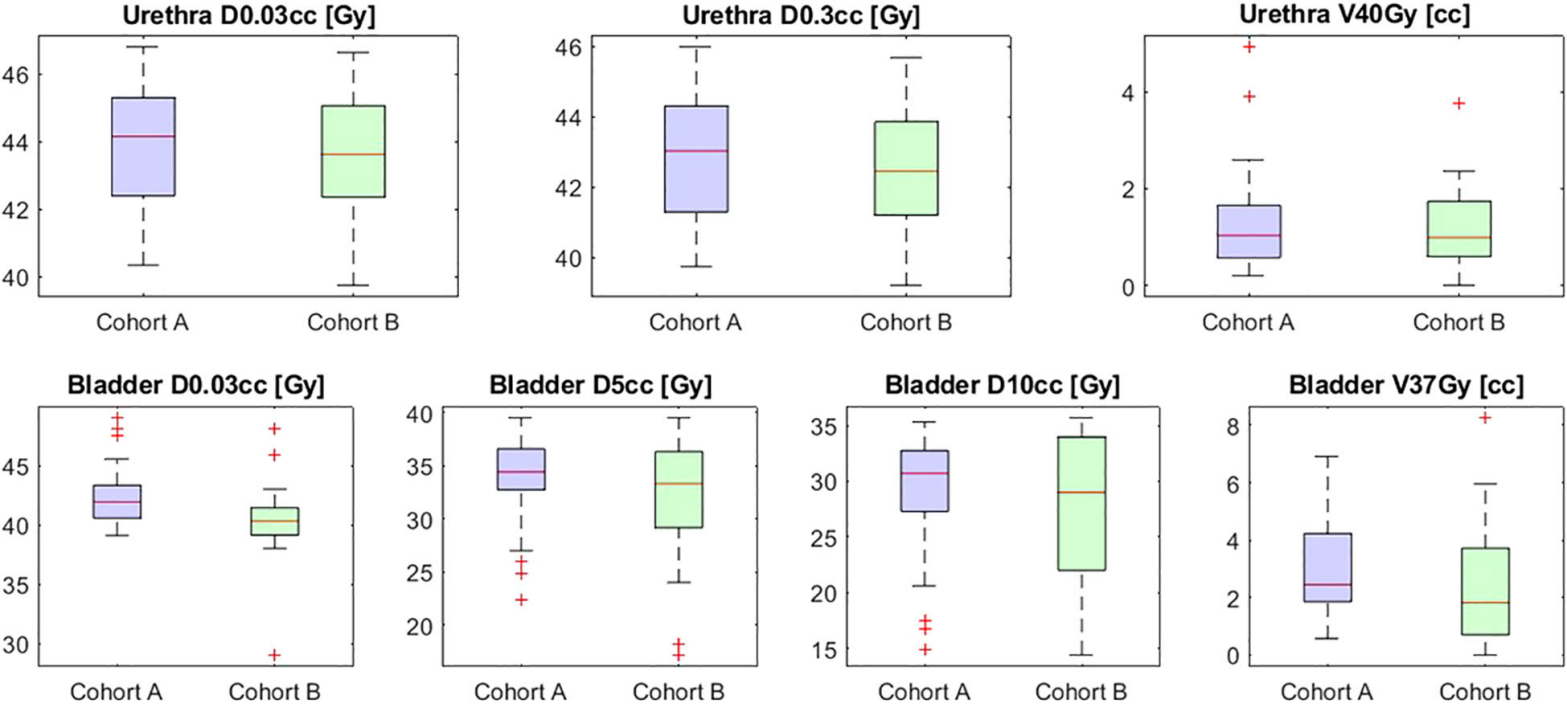
Boxplots of dosimetric parameters for cohorts A and B.

Bladder D0.03cc was significantly higher in cohort A compared to cohort B (41.9 Gy vs 40.2 Gy; p < 0.001). No significant differences were observed in other bladder parameters (D5cc, D10cc, and V37Gy). Similarly, urethral parameters such as D0.03cc (44.2 Gy vs 43.6 Gy; p = 0.49) and V40Gy (1.0 cc vs 0.99 cc; p = 0.94) showed no significant differences between cohorts. These results are summarized in **Table 2** and **Figure 2** below.

**Table 2:** Summary of dosimetric parameters for cohorts A and B.

Dosimetric Parameters	Cohort A	Cohort B	p-value
*D0.03cc Urethra*	44.2 Gy	43.6 Gy	0.49
*D0.3cc Urethra*	43.0 Gy	42.5 Gy	0.68
*V40Gy Urethra*	1.0cc	1.0cc	0.94
*D0.03cc Bladder*	41.9 Gy	40.2 Gy	<0.001
*D5cc Bladder*	34.4 Gy	33.3 Gy	0.16
*D10cc Bladder*	30.7 Gy	29.0 Gy	0.76
*V37Gy Bladder*	2.4cc	1.8cc	0.051

## Discussion

Overall, 62% of patients in this study experienced a MID in urinary quality of life post-SBRT. However, median urinary quality of life returned to baseline at 12 months follow-up among these patients, indicating their recovery as time from treatment passed.

The PACE-B trial has established the non-inferiority in efficacy of SBRT compared to moderately hypofractionated regimen. Nonetheless, concerns persist regarding acute toxicity following SBRT, as highlighted in that study (1). Subjective perception of acute toxicity remains an important consideration in QOL. Notably, although PACE-B shows increased acute urinary toxicity, our cohort did not exhibit sustained self reported worse urinary symptoms. This may suggest that low grade toxicity may be well tolerated by patients and may not impact their quality of life significantly.

This study identifies bladder D0.03cc as a possible dosimetric predictor of decline in urinary QoL in patients undergoing prostate SBRT with IPB. Patients who experienced an MID in IPSS had significantly higher bladder D0.03cc, consistent with prior studies associating high-dose bladder exposure with worse acute urinary symptoms (2, 13). A literature review by Wang et al. demonstrated the relationship between urinary side effects and increased dose to the bladder and urethra (14). Specifically, Kole et al. reported an increased risk of IPSS-defined urinary flare with a bladder D12.7% > 33.5 Gy (15). The results of our study confirm the association demonstrated in previous literature and highlight the importance of limiting bladder dose during treatment planning to mitigate long-term adverse outcomes.

Conversely, urethral dose parameters did not show significant differences between cohorts, suggesting that current dose constraints for the urethra are adequate even with dose escalation. These results align with evidence supporting the safety of selective partial gland dose escalation with SBRT (7, 8).

The longitudinal assessment of IPSS provides valuable insights into the temporal evolution of QoL, showing that most patients’ urinary function improves over time despite the acute decline. This is ultimately consistent with earliest multi-institutional series (16). Interestingly, patients who experienced MID also had lower initial IPSS, suggesting that the acute changes in their QOL were perceived to be more pronounced from the baseline. Of note, patients in Cohort A were treated with ADT in greater proportion than Cohort B which may partly explain their lower initial IPSS score. Interestingly despite the higher rates of ADT in cohort A, they experienced MID in IPSS score in larger proportions.

Ultimately as previously observed in other disease sites patients with transient declines in their urinary symptoms may benefit from interventions such as pelvic floor exercises (17) or as commonly practiced in high volume centers such as ours with early pharmacologic intervention or prophylaxis with alpha blockers (9–11, 18).

Although bladder D0.03cc was identified as a statistically significant dosimetric predictor of symptom change, the relatively modest dose difference between cohorts suggests that this metric alone may not represent a true threshold effect, but rather reflects part of a more complex multifactorial process.

Additionally, in a larger cohort of patients comparing those who received an IPB to those who did not, we did not observe a consistent correlation between urethral or bladder dosimetric parameters and worsening acute or late urinary toxicity. In fact, no grade 3 or higher acute urinary toxicities were observed during this period (18). These findings suggest that careful planning and adherence to urethral and bladder constraints can mitigate potential dose-related toxicity, even in the context of focal dose escalation.

Limitations in this study, such as the retrospective design and single-institution cohort, warrant caution in generalizing the findings. Due to the small number of patients, limited statistical modeling can be conducted. Finally, IPSS has been widely used to assess urinary symptoms in numerous prostate cancer studies and remains the most validated and standardized tool for evaluating lower urinary tract symptoms in this setting. To our knowledge, there is no established evidence indicating that alternative instruments provide superior sensitivity or specificity in detecting changes related to bladder dose. Nonetheless, future studies incorporating complementary questionnaires may provide additional granularity in assessing voiding dysfunction.

## Conclusion

Urinary QoL life changes appear transient and acceptable in the majority of patients treated with partial gland dose escalation as outlined in this study. Bladder D0.03cc emerges as the only statistically significant parameter influencing urinary QoL in prostate SBRT with IPB. Careful dosimetric planning remains essential to achieve optimal therapeutic outcomes while preserving QoL.

## Data Availability

The datasets presented in this article are not readily available because of institutional regulations governing data use agreement. Requests to access the datasets should be directed to corresponding author.
